# Diagnosis of Guillain–Barré syndrome in children and validation of the Brighton criteria

**DOI:** 10.1007/s00415-017-8429-8

**Published:** 2017-03-01

**Authors:** Joyce Roodbol, Marie-Claire Y. de Wit, Bianca van den Berg, Vivienne Kahlmann, Judith Drenthen, Coriene E. Catsman-Berrevoets, Bart C. Jacobs

**Affiliations:** 1 Department of Neurology, Erasmus MC-Sophia Children’s Hospital, University Medical Center Rotterdam, PO box 2040, 3000 CA Rotterdam, The Netherlands; 2 Paediatric Neurology, Erasmus MC-Sophia Children’s Hospital, University Medical Center Rotterdam, Rotterdam, The Netherlands; 3 Immunology, Erasmus MC-Sophia Children’s Hospital, University Medical Center Rotterdam, PO box 2040, 3000 CA Rotterdam, The Netherlands; 4 Clinical Neurophysiology, Erasmus MC-Sophia Children’s Hospital, University Medical Center Rotterdam, Rotterdam, The Netherlands; 5 Department of Neurology, St. Elisabeth Hospital, Tilburg, The Netherlands

**Keywords:** Guillain–Barré syndrome, Pediatrics, Brighton criteria, Cerebrospinal fluid, Nerve conduction study

## Abstract

To describe the key diagnostic features of pediatric Guillain–Barré syndrome (GBS) and validate the Brighton criteria. Retrospective cohort study of all children (<18 years) diagnosed with GBS between 1987 and 2013 at Sophia Children’s Hospital, Erasmus MC, Rotterdam. Clinical information was collected and the sensitivity of the Brighton criteria was calculated. 67 children (35 boys) were included, with a median age of 5.0 years [interquartile range (IQR) 3.0–10.0 years]. Bilateral limb weakness was present at hospital admission in 93% of children, and at nadir in all patients. Children presented with tetraparesis in 70% or with paraparesis in 23%. Reduced reflexes in paretic limbs were observed at hospital admission in 82% and during follow-up in all children. The progressive phase lasted median 6 days (IQR 3–8 days) and less than 4 weeks in all children. A monophasic disease course was seen in 97%, including 5 children with a treatment-related fluctuation. Two children had a later relapse at 9 weeks and 19 weeks after onset. 77% of the children showed an elevated protein level in CSF. Nerve conduction studies showed evidence for a poly(radiculo)neuropathy in 91% of the children. 46 children had a complete data set, the sensitivity of the Brighton criteria level 1 was 72% (95% CI 57–84) and 96% (95% CI 85–99) for level 2 and 98% (95% CI 88–100) for level 3. The majority of the pediatric GBS patients presented in this cohort fulfilled the current diagnostic criteria.

**Electronic supplementary material:**

The online version of this article (doi:10.1007/s00415-017-8429-8) contains supplementary material, which is available to authorized users.

## Introduction

The Guillain–Barré syndrome (GBS) is a clinical diagnosis, supported by the results of the cerebrospinal fluid (CSF) and nerve conduction studies (NCS) [1]. Recognition of GBS is important to start treatment and monitoring as soon as possible. Accurate diagnostic criteria for GBS are also required to determine background incidence rates and to conduct vaccine safety studies. For this purpose, criteria for GBS were developed in 1978 by the National Institute of Neurological and Communicative Disorders and Stroke (NINDS) and updated in 1990 [2]. In response to the H1N1 vaccination campaign in 2009 and its possible relation with GBS, new case definitions for GBS were developed by the Brighton Collaboration, an international collaboration sponsored by the World Health Organization to improve vaccine safety monitoring [3]. Previous studies have validated the Brighton criteria in cohorts of adult patients with GBS from South Korea and The Netherlands [4, 5]. The Brighton criteria also require validation for GBS in children, which may differ considerably from GBS in adults [6].

In 2011 the Brighton criteria were validated in children from India, but the clinical presentation of GBS in India is not the same as in a Western country. Therefore, in this study, we describe in detail the clinical presentation and course of GBS in children, focussing on the key diagnostic characteristics, and validate the Brighton criteria for pediatric GBS.

## Patients and methods

Medical files and discharge letters from all children (<18 years) diagnosed with GBS at Sophia Children’s Hospital, Erasmus MC, Rotterdam, The Netherlands, between 1987 and 2013, were reviewed retrospectively. Part of this cohort has been described previously for different purposes [6]. The revised version of the NINDS diagnostic criteria from 1990 [2] was used as guideline for the diagnosis. Patients with Miller Fisher syndrome, acute onset chronic inflammatory demyelinating polyneuropathy (CIDP) or other neurological diseases or comorbidity influencing the GBS diagnosis were excluded. Data were collected regarding age, sex, preceding events, onset of weakness, neurological signs and symptoms at hospital entry, clinical course, and results from CSF examination and NCS. Severity of the disease at nadir was defined by the highest GBS disability score (Table 1) [7].

**Table 1:** Neurological deficits at admission and nadir in 67 children with GBS

Demography	Presentation
Male/female ratio	35/32
Age at admission (years)^a^	5 (IQR 3–10, range 0–16)
Antecedent events	
Diarrhea	40% (25/64)
Upper respiratory tract infection	41% (27/66)
Vaccination	8% (5/67)

	Admission	Nadir
Neurological signs and symptoms		
Cranial nerve deficits	53% (32/61)	60% (39/65)
Sensory deficits	30% (12/40)	40% (19/48)
Pain	73% (47/64)	84% (54/64)
Bilateral weakness	93% (55/59)	100% (65/65)
Tetraparesis	70% (40/57)	88% (57/65)
Paraparesis (of the legs)	23% (13/57)	11% (7/65)
Decreased reflexes in weak limbs	82% (45/55)	100% (62/62)
Autonomic dysfunction	12% (8/67)	53% (35/66)
Blood pressure fluctuations	6% (4/66)	35% (23/65)
Cardiac dysrhythmia	3% (2/67)	15% (10/66)
Bladder dysfunction	6% (4/65)	25% (16/64)
GBS disability score^b^		
2	35% (23/66)	8% (5/67)
3	26% (17/66)	16% (11/67)
4	36% (24/66)	51% (34/67)
5	3% (2/66)	24% (16/67)
6	0% (0/66)	2% (1/67)

*2* able to walk 10 m unaided, unable to run, *3* able to walk 10 m with aid, *4* Bedridden or chairbound, *5* requiring assisted ventilation, *6* deceased

^a^Median (interquartal range and full range)

^b^GBS disability score

Symmetrical weakness was defined as the absence of difference in muscle weakness in major limb muscle groups on the left versus the right side.

A clinical fluctuation was previously defined as an improvement or stabilization longer than 1 week followed by secondary deterioration of at least one grade in the GBS disability score [8, 9]. A treatment-related fluctuation (TRF) was defined previously as a clinical fluctuation due to the transient effect of the treatment that usually occurs within 8 weeks after start of treatment [8].

CSF was examined for protein level and for cell count. All results of the NCS were reviewed by a clinical neurophysiologist (JD) and were considered supportive of the clinical diagnosis if consistent with the criteria for acute inflammatory demyelinating polyneuropathy (AIDP), acute motor axonal neuropathy (AMAN), acute motor axonal sensory neuropathy (AMSAN) or inexcitable nerves [10]. An equivocal electrophysiological result not meeting the criteria for one of these subtypes, but still consistent with the diagnosis GBS, was recorded separately [5].

The Brighton criteria consist of four levels of diagnostic certainty. Level one has the highest diagnostic certainty; these patients fulfil all diagnostic criteria. Level 4 has the lowest diagnostic certainty, these patients do not fulfil the criteria of level 3 but all other diagnoses are excluded. The diagnostic criteria needed to fulfil each level are shown in online only Table 1. All patients in this study were classified according to the Brighton criteria. This was done for the entire cohort and also in a subgroup of patients in whom all clinical information regarding the six key diagnostic features were present. Sensitivity of the Brighton criteria was calculated for the levels 1, 2 and 3.

The study was approved by the medical ethical review committee of the Erasmus MC.

### Statistical analysis

Continuous data were presented as means and standard deviations if normally distributed, and as medians and interquartile ranges (IQR) when not normally distributed. Categorical data were presented as proportions. Shapiro–Wilk test was used to define if the data were normally distributed. Continuous data were compared with *t* test if normally distributed and with Mann–Whitney *U* test if not normally distributed. Proportions were compared using the Chi-square or Fisher exact test. Correlations between categorical data were tested using the Spearman correlation. SPSS Statistics 20.0 was used for the statistical analyses. A two-sided *p* value <0.05 was considered to be statistically significant.

## Results

The clinical features of 67 children diagnosed with GBS included in the study are shown in Table 1. The key diagnostic characteristics important for the Brighton criteria are described in more detail.

### Muscle weakness

At admission all children presented with symmetrical limb weakness, except four cases (6%). These four children without weakness initially presented with different symptoms or signs, including neck stiffness, facial weakness and limb muscle pain. In the following days, all developed a symmetrical tetraparesis and were diagnosed with GBS. At hospital admission, 13 (23%) children presented with weakness restricted to the legs. During the course of the disease, six of them developed additional weakness of the arms. The remaining seven (11%) children showed persistent paraparesis of the legs during follow-up, although three of them developed reduced reflexes of the arms.

### Reflexes

At admission, most children had decreased reflexes in weak limbs (Table 1). One child initially had hyperreflexia in weak limbs with plantar reflexes and a normal MRI of cerebrum and myelum, excluding transverse myelitis. This patient developed hyporeflexia during the course of the disease.

### Course of the disease

All children reached nadir within 28 days of onset of weakness and 43 (66%) children already within 1 week. 65 children (97%) had a monophasic disease course. Five (8%) children had a clinical fluctuation within 8 weeks of onset of weakness, which were interpreted as TRF but considered compatible with a monophasic disease course. Two of these children received a second course of IVIg because of the severity of the deterioration. Two (3%) children had a clinical fluctuation more than 8 weeks after onset of weakness. One child deteriorated at day 59 but the weakness was milder than the first episode. The other child had a clinical fluctuation at day 137 during a *Shigella* gastro-enteritis. Both children recovered spontaneously and no further fluctuations occurred.

One child had a relapsing GBS with two recurrences at 3 and 23 years after the first episode. She was treated with a course of IVIg after each episode with a good clinical response and was stable without residual symptoms between these episodes. One patient died during the course of the disease due to severe autonomic dysfunction.

### Treatment

In this cohort, only 6 children (9%) did not receive specific treatment for GBS. The majority of the children received either IVIg alone (*N* = 49, 77%) or plasma exchange alone (*N* = 1, 2%) or a combination of IVIg and prednisone/methylprednisolone (*N* = 8, 13%). This last category of patients participated in the randomized controlled trial comparing IVIg and methylprednisolone (MP) versus IVIg and placebo (Koningsveld et al. 2004) [11]. Some patients received IVIg and MP shortly after finalizing the trial considering that this combination was related with better outcome after adjustment for age and GBS disability score. One of these eight children had increased intracranial pressure during the acute phase of GBS for which he received IV steroids.

### CSF examination

**Fig. 1:**
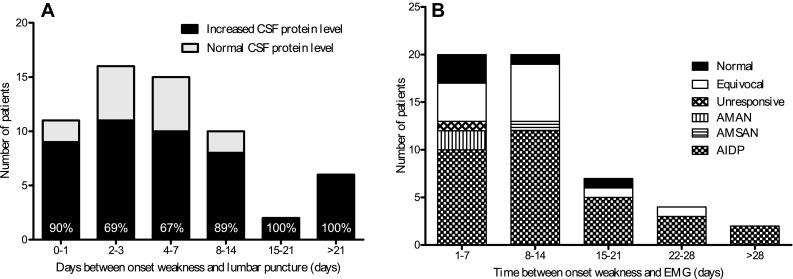
Diagnostic test results. **a** Frequency of increased protein levels in CSF in children with GBS. Reference values CSF protein in children used in Sophia children’s hospital: 1–3 months: 0.24–0.65 g/L, 3–6 months: 0.23–0.37 g/L, 6–12 months: 0.17–0.35 g/L, 1–10 years: 0.16–0.31 g/L, 10–18 years: 0.24–0.49 g/L. %, the percent of patients with an increased CSF protein level. **b** Results nerve conduction studies in children with GBS

In 62 (93%) patients a lumbar puncture was performed, but only in 57 (85%) patients the results on both cell count and protein level were available. The interval between onset of weakness and lumbar puncture was median 4 days (IQR 2–8 days, range 0–26 days). A mild pleocytosis was observed in 27 children (47%); the cell count ranged between 5 and 10 leukocytes/μl in 15 children, 11 and 20 leukocytes/μl in 10 children and 21 and 50 leukocytes/μl in 3 children. Two children showed an increased cell count with 54 and 60 leukocytes/μl, but both children may have had a traumatic puncture; no other diagnosis was made. CSF protein level was increased in 46 (77%) of the patients (Fig. 1a). Of the children with a raised CSF protein level, at least four had a traumatic puncture. Most CSF samples obtained more than 1 week after onset of weakness showed an increased protein level.

### Nerve conduction studies

In 53 (79%) patients data from NCS were available for the current study (Fig. 1b). The median number of days between onset of weakness and NCS was 9 days (IQR 5–14 days, range 1–37 days). In 48 (91%) children, the NCS showed evidence for the presence of a poly(radiculo)neuropathy and supported the diagnosis GBS. In the remaining five (9%) children, the NCS were normal and not repeated. AIDP was the predominant subtype present in 53 children (60%), followed by AMAN in 2 children (4%), both AMSAN and unresponsive nerves in one child (2%) In 12 children (23%), the abnormal NCS were equivocal and could not be further classified. Of the 7 children with persistent paraparesis, NCS of the arms was performed in 2 children, and in both patients the results indicated a demyelinating polyneuropathy of both arms and legs. The electrophysiological subtype was not related to the interval between onset of weakness and performing the NCS.

### Brighton classification

The classification according to the Brighton criteria depends on the completeness of the data regarding the key diagnostic characteristics. Therefore, the criteria were validated separately for the subgroup of patients with a complete dataset and for the total group of patients (Online Resource Table 2). In the 46 children with a complete dataset, 33 children reached level 1, 11 children reached level 2, one child reached level 3 and one child reached level 4. From the children who did not reach level 1, 8 children (17%) either had a normal CSF protein level or normal NCS, 3 children (7%) had both a normal CSF protein level and a normal NCS. The child who reached level 3 had more than 50 leukocytes in CSF. The patient who only reached level 4 did not have a monophasic disease course. The sensitivity was 72% (95% CI 57–84) for level 1, 96% (95% CI 85–99) for level 2 and 98% (95% CI 88–100) for level 3. Patients with various Brighton levels did not differ regarding age, sex, preceding event, disease severity and outcome. As expected, the Brighton criteria had a lower sensitivity in the whole group of patients because of missing data (Online Resource Table 2).

## Discussion

In the current study, we described in detail the variation in key clinical characteristics in 67 children with GBS. The far majority showed a rapidly progressive flaccid tetraparesis with reduced reflexes reaching nadir within 4 weeks, followed by slow recovery without relapses. The diagnosis was confirmed in most cases by the presence of an increased protein level in CSF and findings compatible with a poly(radiculo)neuropathy in NCS. In a minority of children, the presentation differed from this prototypic form. First, 13 (23%) children presented with paraparesis of the legs and seven (11%) showed a persistent paraparesis during the entire course of disease. In none of these patients, there was evidence for myelum involvement based on clinical examination, MRI of the spine (conducted in 2 of 7 cases), or clinical re-examination during follow-up. A similar paraplegic variant of GBS has been reported previously in adult patients (18). Second, CSF protein level was normal in 14 (23%) children, and a CSF pleocytosis between 5 and 50 leukocytes/μl was observed in 28 (49%) children. Third, results of NCS were equivocal in 12 (23%) and normal in 5 (9%) children, implying that in one-third the electrophysiological subtype could not be defined in a single NCS using current criteria. Additional diagnostic work-up and a follow-up of at least 6 months in these atypical cases revealed no alternative diagnosis, indicating that these variations are part of the spectrum of phenotypes within the diagnosis of GBS. Children with a complete dataset reached Brighton level 1 in 72%, and at least level 3 in 98%, indicating that the Brighton criteria have a high sensitivity for the diagnosis of GBS in children. These results show that children usually present with the classic symptoms of GBS and fulfill the current diagnostic criteria.

Accuracy of the Brighton criteria developed for vaccine safety monitoring is especially relevant for children who are frequently exposed to vaccinations. Two previous studies have reported on the sensitivity of the Brighton criteria for GBS in children [4, 12]. A study from India was based on the national polio surveillance program in children (<15 years) and selected 79 (11%) patients with a full diagnostic workup from an original population of 718 children diagnosed with GBS [12]. This study showed a comparably high frequency of patients reaching level 1 (62%) or at least level 3 (86%), and a similar frequency of patients with normal CSF results (16%) and equivocal NCS (29%). The authors indicated that the investigated population in their study was likely biased towards more severe cases who more frequently get a full diagnostic work-up. In a study from South-Korea, none of 18 children reached level 1, but all reached level 2 or 3 [4]. Compared to a previous study in adult patients from The Netherlands, the Brighton criteria are more sensitive for the diagnosis of pediatric GBS [5]. Only 61% of adult patients reached level 1, compared to 72% of children in the current study. This difference is almost fully explained by the lower frequency of an increased protein level in CSF in adults compared to children, despite the fact that the timing of the lumbar puncture in adults and children was similar. A limitation of all studies investigating the performance of the Brighton criteria in pediatric GBS, including ours, was the retrospective design and the influence of missing data.

Additional investigations of CSF and NCS were not performed in ten children (15%). Most frequently, these studies were not conducted because the patient had a mild form of GBS or no alternative diagnosis was suspected. Physicians may also be more reluctant to perform invasive or painful investigations in children than adults. Less-invasive techniques than lumbar puncture and NCS may be considered to confirm the diagnosis of GBS. Recently, gadolinium-enhanced magnetic resonance imaging of the nerve roots was reported to be equally accurate as NCS [13], and lumbar puncture [14]. MRI may be especially valuable in centers with limited pediatric neurophysiological expertise. Other diagnostic techniques potentially useful in children are the compound muscle action potential scan (CMAP scan) [15] and nerve ultrasound [16]. To further improve the diagnostic criteria for GBS, the specificity needs to be defined in children with similar clinical presentations as GBS but an alternative diagnosis. In addition, prospective studies are required including patients with the full spectrum of subforms of GBS. The Brighton criteria were developed primarily for surveillance and vaccine safety studies rather than for clinical decision-making in the diagnostic work-up in clinical practice in individual patients. Development of protocols for routine diagnostic work-up and criteria for early diagnosis would support the early diagnosis of GBS in children.

## Electronic supplementary material

Below is the link to the electronic supplementary material.

Supplementary material 1 (PDF 30 kb)

Supplementary material 2 (PDF 17 kb)
