# Supplementary material for: Diagnosis of Guillain–Barré syndrome in children and validation of the Brighton criteria
Source: J Neurol. 2017 Mar 1;264(5):856–61. doi: 10.1007/s00415-017-8429-8 (PMC5413522; doi:10.1007/s00415-017-8429-8)
Supplement: Supplementary file 1 — Supplementary material 1 (PDF 30 kb) [file 415_2017_8429_MOESM1_ESM.pdf]

Online Resource table 1: Summary of the Brighton case definitions for Guillain-Barré syndrome.

	Level of diagnostic certainty			
**Diagnostic criteria**	1	2	3	4
Bilateral and flaccid weakness of limbs	+	+	+	+/-
Decreased or absent deep tendon reflexes in weak limbs	+	+	+	+/-
Monophasic course and time between onset-nadir 12 hr-28 days	+	+	+	+/-
CSF cell count < 50/µl	+	+ [a]	-	+/-
CSF protein concentration > normal value	+	+/- [a]	-	+/-
NCS findings consistent with one of the subtypes of GBS	+	+/-	-	+/-
Absence of alternative diagnosis for weakness	+	+	+	+

+ = present; - = absent; +/- = present or absent; CSF = cerebrospinal fluid; NCS = nerve conduction studies; GBS = Guillain-Barré syndrome

[a] If CSF is not collected or results not available, nerve electrophysiology results must be consistent with the diagnosis GBS.

(Table derived from Fokke et al.Brain. Jan, 2014) [5]
