# Supplementary material for: Diagnosis of Guillain–Barré syndrome in children and validation of the Brighton criteria
Source: J Neurol. 2017 Mar 1;264(5):856–61. doi: 10.1007/s00415-017-8429-8 (PMC5413522; doi:10.1007/s00415-017-8429-8)
Supplement: Supplementary file 2 — Supplementary material 2 (PDF 17 kb) [file 415_2017_8429_MOESM2_ESM.pdf]

Online Resource table 2: Classification of children with GBS according to Brighton criteria.

**Levels Brighton criteria**	**Data complete** (N=46)	**All patients** (N=67)
**Level 1**	**72% (33)**	**49% (33)**
AIDP, AMAN, AMSAN, unresponsive	59% (27)	40% (27)
Equivocal	13% (6)	9% (6)
**Level 2**	**24% (11)**	**34 % (23)**
Normal CSF protein level or normal NCS[a]	17 % (8)	12 % (8)
Normal CSF protein level and normal NCS[a]	7 % (3)	4 % (3)
NCS or lumbar puncture not performed[a]	-	9 % (6)
Normal CSF protein level and no NCS performed[a]	-	2% (1)
CSF or NCS missing[a]	-	6 % (4)
Normal CSF protein level and missing NCS [a]	-	2 % (1)
**Level 3**	**2 % (1)**	**6 % (4)**
EMG and lumbar puncture not performed[b]	-	3 % (2)
CSF cell count >50[b]	2 % (1)	2 % (1)
Normal NCS and no lumbar puncture performed[b]	-	2 % (1)
**Level 4**	**2 % (1)**	**10 % (7)**
Reflexes missing[c]	-	3% (2)
No monophasic disease[c]	2% (1)	3 % (2)
Reflexes missing, no NCS performed and CSF cell count > 50[c]	-	2 % (1)
Reflexes and NCS missing and normal protein level in CSF[c]	-	2 % (1)
Course of the disease unknown and no NCS performed[c]	-	2 % (1)

GBS patients were classified according to the case definitions of the Brighton criteria. We separately shown the results of the children with a complete dataset (N=46) and all patients (N=67). In level 1 we showed the results of the NCS dividing AIDP, AMAN, AMSAN and unresponsive from equivocal.

[a] Reasons why patients did not reach level 1

[b] Reasons why patients did not reach level 2

[c] Reasons why patients did not reach level 3
